# Cold tolerance identification of nine *Rosa* L. materials and expression patterns of genes related to cold tolerance in *Rosa hybrida*

**DOI:** 10.3389/fpls.2023.1209134

**Published:** 2023-06-27

**Authors:** Hongli Wang, Xi Cheng, Qiyu Shi, Jie Xu, Dongliang Chen, Chang Luo, Hua Liu, Li Cao, Conglin Huang

**Affiliations:** ^1^ Beijing Engineering Research Center of Functional Floriculture, Institute of Grassland, Flowers and Ecology, Beijing Academy of Agriculture and Forestry Sciences, Beijing, China; ^2^ College of Agriculture, Yanbian University, Yanji, China

**Keywords:** *Rosa* L., cold tolerance, RNA-Seq, qRT-PCR, resource evaluation

## Abstract

Members of the *Rosa* genus have a high ornamental value, but their cultivation area is limited by their sensitivity to cold temperatures. The aim of this study was to evaluate the cold tolerance of a range of *Rosa* materials, and then determine which genes were related to cold tolerance. Nine *Rosa* materials were subjected to a cold treatment. To identify genes related to cold tolerance, *R. hybrida* was treated at −15°C for 10 min, and leaves collected before and after this treatment were collected for RNA-Seq analyses. The transcript profiles of four DEGs (*POD17, NDUFA9, PMA1*, and *b-Amy1*) in *R. hybrida* were determined by qRT-PCR at 0 h, 1 h, 2 h, and 3 h at −15°C. Nine *Rosa* materials were subjected to a cold treatment, and the most cold-tolerant materials were identified as those that showed the lowest levels of electrolyte leakage and the best recovery after 30 d of growth. The most cold-tolerant materials were *Rosa hybrida, Rosa rugosa* ‘Pingyin 12’, and *Rosa rugosa*. In total, 204 significantly differentially expressed genes (DEGs) were identified, of which 88 were significantly up-regulated and 116 were significantly down-regulated under cold conditions. Gene Ontology classification and Kyoto Encyclopedia of Genes and Genomes pathway analyses showed that the DEGs were enriched in 57 pathways, especially starch and sucrose metabolism, phenylpropane biosynthesis, MAPK signaling, fructose and mannose metabolism, and oxidative phosphorylation. By transcriptional analysis, *PMA1*, which was related to H^+^ ATPase activity, was continuously up-regulated, but the transcript levels of *POD17, NDUFA9*, and *β-Amy1* fluctuated during the freezing treatment. This research uncovered scarce cold-resistant materials and layed the foundation for further research on the cold tolerance mechanism of *Rosa* plants and the breeding of cold-tolerant varieties.

## 1 Introduction

There are about 200 species in the *Rosa* genus, of which 82 species are native to China. *Rosa* species are widely distributed in various climate zones around the world (Che et al., 2016). Among them, *Rosa chinensis* Jacq., *Rosa rugosa*, and *Rosa* sp. are the most representative, and are popular ornamental plants worldwide. There are many varieties of *Rosa* species, with rich colors and a strong scent. They not only have a high ornamental value, but are also important raw materials for the production of spices and foods. They also have a high nutritional and medicinal value (Guo et al., 2011). *R. chinensis* Jacq. is an evergreen and semi-evergreen low shrub. It is known as the ‘Queen of Flowers’ and is very popular with consumers. *R. chinensis* can be grown under a wide range of conditions and produces flowers in all four seasons. Its flowers show a diverse array of colors and types. It is an important plant for urban beautification and courtyard greening, but it is not sufficiently cold resistant to grow in northern areas of China. *R. rugosa* is an upright shrub that is not only attractive in its appearance, but also useful for making spices, cosmetics, and food. *R. rugosa* has strong tolerance to drought, saline-alkali soils, low-nutrient soils, and cold and other abiotic stresses, and it is an excellent ornamental plant breeding resource. However, different varieties of *R. rugosa* differ in their degree of cold tolerance. *Rosa* sp. are important wild resources with many excellent shapes and growth forms, beautiful flower colors, diverse flower types, high ornamental value, and strong cold resistance. With the increasing use of *Rosa* plants for planting, as cut flowers, and in products, there is an increasing demand for *Rosa* materials with particular characteristics. Through the joint efforts of breeders all over the world, many new varieties have been bred, but there are still resources that are underutilized and lack in-depth research.

As an important agronomic trait, cold resistance has attracted much attention. Cold temperatures can result in symptoms of wilting, yellowing, or necrosis, and these symptoms are related to changes in the physiological and biochemical functions of plant cells (Ruelland and Zachowski, 2010). The commonly used methods for identifying cold tolerance include electrolyte leakage, electrical impedance spectroscopy, chlorophyll fluorescence, differential thermal analysis, and whole plant freezing tests (Janská et al., 2010). Of those, the electrolyte leakage method is more accurate, requires less equipment and fewer samples, and is simple to operate. Thus, it is the most widely used.

Plants respond to low-temperature stress through two main processes: low temperature perception and signal transduction (Li, 2017). Low temperatures directly inhibit metabolism-related enzyme activity and alter gene expression in plants. Under low temperature, plants undergo extensive changes in their physiology and metabolism, including changes in cell membrane phase and permeability, and in the concentrations of hormones and cryoprotectant molecules (such as soluble sugars and proline). These changes result from changes in the expression of related genes. Recent research on the plant response to low temperatures has focused on the ICE1-CBF-COR transcriptional cascade signaling pathway (Tomashow, 1999; Chinnusamy et al., 2003). Under low-temperature stress, there are increased transcript levels of many genes encoding transcription factors, including ICE1 and CBF. ICE1 is an upstream transcription factor that regulates the transcription of *CBF* under low temperature. It is a constitutively expressed gene whose product is phosphorylated under cold stress. The CBF transcription factor activates the expression of many downstream COR (Li, 2017), thereby playing a very important role in cold adaptation. In addition to transcription factors, proteases are related to cold tolerance in plants. Teige et al. (2004) found that *MKK2* in the *Arabidopsis* MAPK signaling pathway is activated under cold and salt stress conditions, and regulates the expression of downstream genes involved in the low temperature response. Although previous studies have discovered many genes related to cold tolerance in plants, less is known about the mechanism of the perception of cold temperatures. It is important to explore this topic to breed new, cold-tolerant varieties of economically important plants.

RNA-Seq and analysis techniques have been applied in many fields, such as biology and medicine. RNA-Seq analyses can reveal new genes, gene transcripts with low abundance, and alternative splicing variants. Data generated in RNA-Seq analyses can provide important information about metabolic pathways and gene families involved in various responses, and are useful for evolutionary analyses (Song, 2016). Because RNA-Seq datasets are very large, they can enrich the genetic information available for a plant species. They can provide a large amount of related ESTs information, making it possible to detect changes in gene expression levels, and allowing for the discovery of new functional genes. The information generated in RNA-Seq analysis lays the foundation for subsequent gene cloning and the development of molecular markers such as SNP and simple sequence repeat markers, which are useful for mapping and breeding. RNA-Seq can reveal patterns of gene expression in specific tissues or cells, and reveal previously unknown small RNAs (Ke, 2016). In recent years, RNA-Seq technology has been widely used to study the mechanism of cold tolerance in many plants, such as *Arabidopsis thaliana* (Lee et al., 2005), wheat (Gulick et al., 2005), rice (Zhang et al., 2012), corn (Sobkowiak et al., 2014), and cassava (An et al., 2012). Analyses of cold-treated plant materials have generated a wealth of transcriptome data and revealed many transcription factors related to low-temperature signal transduction and the low-temperature stress response. This has established a baseline for research on other crops, and provided a way to unravel the complex mechanisms that underly tolerance to cold temperatures in plants.

Low-temperature stress is one of the main factors restricting the cultivation and production of *Rosa.* Low temperatures in winter negatively affect the quality of *Rosa* materials, resulting in shorter branches, smaller leaves, deformed petals, color degradation, and a prolonged growth period. This can cause substantial losses for producers (Zhu, 2017). In plants, cold resistance is the result of the combined effects of genetic factors and environmental factors (He, 1995), and comprehensive research using multiple methods from multiple perspectives is required to explore its mechanisms in detail. Low temperature stress damages the structure and function of plant biofilms, and affects the activity and abundance of various enzymes involved in physiological and biochemical processes. This leads to metabolic disorders that negatively affect plant growth, development, and yield, and limit plant cultivation and distribution. Screening to identify cold-tolerant varieties has been conducted for a variety of plants, such as *Solanum lycopersicum*, *Rhododendron simsii*, *Camellia oleifera*, and *Zoysia japonica*. The identification of cold-tolerant *Rosa* materials is also advancing. At present, low temperatures in winter limit the geographical distribution of *Rosa*. Therefore, it is of great significance to identify cold-resistant *Rosa* materials to enrich the resources of high-quality cold-resistant varieties, and to guide people in the use of those varieties. The identification of cold-resistant *Rosa* materials will be useful to provide a scientific basis for the breeding and cultivation of cold-resistant resources, and to expand the cultivation and distribution range of *Rosa* plants.

To date, research on *Rosa* plants in China and elsewhere has focused on disease resistance and stress resistance mechanisms, breeding, flower aroma and essential oil composition, genetic relationships and classification of varieties, improvements in cultivation technology and cultivated varieties, cut flower preservation, wild resource distribution and ecology, essential oil extraction, factors contributing to endangerment, and protection strategies. However, few studies have focused on physiological and biochemical characteristics related to the cold tolerance of roses. In previous studies, the most and least cold-resistant genotypes were screened by evaluating the cold resistance of 17 different genotypes of courtyard roses. It was found that sucrose and oligosaccharides were related to the cold resistance of roses (Lin et al., 2019a). A study on the seasonal changes in the cold hardiness and carbohydrate metabolism of four garden *Rosa* cultivars revealed differences in the transcript levels of genes linked to dehydrin and carbohydrate metabolism during cold acclimation between the more cold-hardy ‘Dagmar Hastrup’ and the less cold-hardy ‘Chandos Beauty’ (Lin et al., 2019b).

In this study, we subjected *Rosa* materials to a low-temperature treatment and observed changes in certain biological characteristics and physiological indicators to evaluate their cold resistance. Then, by sequencing the leaf transcriptomes of a cold-resistant material before and after a low-temperature treatment, we identified DEGs related to cold resistance. We then analyzed the transcript profiles of four genes potentially involved in cold resistance during a 3-h cold treatment at −15°C using quantitative PCR (qPCR). The aims of this study were to select the varieties with higher cold tolerance from nine materials, to identify genes related to cold tolerance, and to explore the molecular mechanism of cold resistance of *Rosa*. The results of this study lay the foundation for the selection of cold-tolerant *Rosa* materials, the selection and manipulation of cold-tolerance related genes, and the breeding of new cold-tolerant varieties.

## 2 Materials and methods

### 2.1 Evaluation of cold tolerance of nine *Rosa* materials

#### 2.1.1 Plant materials and low-temperature treatment

The nine *Rosa* materials were *R. hybrida*, *R. rugosa* ‘Yilanxiao’, *Rosa maximowicziana*, *R. rugosa* ‘Pingyin 12’, *R. rugosa*, *Rosa multiflora*, *Rosa multiflora hybrida*, *Rosa wichurana* ‘Dian hong’, and *Rosa wichuraiana.* All of these materials were provided by the farm of Beijing Academy of Agriculture and Forestry Sciences. The annual semi-lignified branches of biennial plants were cut to a length of about 10 cm, retaining two to three leaves, and the cuttings were made in a matrix seedbed of grassy soil and vermiculite (1:1) with sprinkler irrigation facilities. At about 40 d after cutting, the rooted cuttings were planted in pots and cultivated under open air conditions, with watering once every 2–3 d. After further 55 days of growth, the cuttings with good growth and high consistency in the material were selected for low temperature treatment.

For each variety, 16 rooted cuttings with strong, consistent growth were selected for the low-temperature treatment (4°C for 2 d, then 0°C for 4 d; light intensity of 350 lux; 12 h light/12 h dark photoperiod; and relative humidity of 70%–80%). After 0 d, 2 d, 4 d, and 6 d of the low-temperature treatment, six to eight mature leaves were collected from each material to measure REC using the method described by Campos et al. (2003). The initial conductivity (R1) at room temperature was measured using a thunder magnetic conductivity meter (DDSJ-308F, Shanghai, China). The pre-treated leaves were then placed in a boiling water bath for 30 min, and the conductivity (R2) was measured after cooling to room temperature, The leaves without low-temperature conditioning were used as control conductivity (R0). Relative conductivity was calculated as follows:


REC/%=(R1−R0)/(R2−R0)*100

After 6 d of the low-temperature treatment, photographs were taken to record changes in morphology, and then four treated plants of each variety were placed in an ultra-low temperature freezer at −15°C for 3 h. At 0 h, 1 h, 2 h, and 3 h, three leaves were selected from the same positions on three plants of *R. hybrida*, wrapped in aluminum foil, placed in liquid nitrogen, brought back to the laboratory, and stored at −80°C until real-time fluorescence quantitative PCR analysis. Finally, all the materials treated at −15°C were allowed to recover in a greenhouse at a constant temperature (24°C) for 30 d with watering once every 4–6 d. These materials were photographed at intervals to record their growth and recovery.

#### 2.1.2 Cold tolerance data analysis

*Cynodon dactylon*, *Vitis vinifera* and *Rosa* plants have evaluated the cold tolerance of plants by membership function method combined with various physiological and biochemical indexes (Zhang et al., 2007; Li et al., 2011; Deng et al., 2012). Therefore, we used the subordination function method to analyze the REC values of different materials. The materials were sorted according to the subordination function value to give a ranking of their cold tolerance. The larger the subordination function value, the stronger the cold resistance of the material.

The REC is positively correlated with cold tolerance; the formula used to calculate the subordination function value was as follows:


$${\text{U}}_{\text{ij}}=\left({\text{X}}_{\text{ij}}{\text{-X}}_{\text{imin}}\right)/\left({\text{X}}_{\text{imax}}{\text{X}}_{\text{imin}}\right)$$

The REC is negatively correlated with cold tolerance; the formula used to calculate the subordination function value was as follows:


$${\text{U}}_{\text{ij}}=1-\left({\text{X}}_{\text{ij}}{\text{–X}}_{\text{imin}}\right)/\left({\text{X}}_{\text{imax}}{\text{–X}}_{\text{imin}}\right)$$

where i represents a certain material, j is the index, U is the membership grade, X_ij_ is the measured value of the index in a certain material, X_imin_ is the minimum value of the index in material i, and X_imax_ is the maximum value of the index in material i.

### 2.2 Mining of DEGs related to cold tolerance in *R. hybrida*

#### 2.2.1 Samples and cold treatment

We selected three plants of *R. hybrida* that produced new branches during the 30-d recovery period. The new branches were cut off and treated at −15°C until they wilted (about 10 min). Before and after this cold treatment, three leaves were selected from the same position on the plant, wrapped in aluminum foil, frozen in liquid nitrogen, and stored at −80°C until RNA extraction. The names of the leaf samples before and after treatment were as follows:

MGCK_1_15;

MGCK_2_18;

MGCK_3_19;

MGCL_1_12;

MGCL_2_13;

MGCL_3_14;

where MCCK denotes control leaves and MGCL denotes leaves subjected to the cold treatment.

#### 2.2.2 Total RNA extraction and quality testing

Total RNA was extracted using the Takara MiniBEST Plant RNA Extraction Kit (Takara, Dalian, China). The concentration, purity, and integrity of the extracted RNA were tested using a NanoPhotometer^®^ Spectrophotometer (Thermo Fisher, Beijing, China), a Qubit^®^ 2.0 Fluorometer (Thermo Fisher, Beijing, China), and an Agilent 2100 RNA Nano 6000 Assay Kit (Agilent, Beijing, China).

#### 2.2.3 Transcriptome library preparation

After checking the quality of the extracted RNA, mRNA was enriched by the magnetic bead enrichment method, and then fragmentation buffer was added to break the mRNA into fragments of about 200 bp. Then, random primer hexamers were added to synthesize cDNA. A nucleic acid purification kit (AMPure XP beads) was used for the first cDNA purification step. The purified cDNA fragments were subjected to end-repair, the addition sequencing adapters at both ends, and then secondary purification. After screening fragments of a suitable size, PCR amplification was conducted to obtain the final cDNA library.

After the library was constructed, the cDNA concentration was determined using a Qubit 2.0 Fluorometer and then adjusted 1 ng/µL. The insert size of the library was checked using an Agilent 2100 instrument to ensure the reliability of the results. An SYBR real-time fluorescent quantification kit (Bio-Rad, Hercules, CA, USA) was used to perform qPCR to determine the concentration of the constructed library, and to construct a standard curve. When the initial library concentration is >2 nM, sequencing can be performed on the HiSeq 2500 sequencing platform. For these analyses, we used a Bio-Rad CFX 96 fluorescent quantitative PCR instrument and separated the products by electrophoresis on 2% agarose gels. After generating clusters on cBot, the paired-end sequencing program (PE150) was run on the Illumina HiSeq 2500 sequencing platform, generating 100-bp or 125-bp paired-end sequencing reads.

#### 2.2.4 Bioinformatics analyses

Sequencing quality assessment and transcript assembly: First, the original sequencing data were sorted and pre-processed. The data were filtered with TrimGalore software (Bolger et al., 2014) and the bases with a quality value greater than 20 were retained. Secondary screening was conducted to remove fragments shorter than 50 bp in length and those with only one end in paired-end sequencing. Because there was no reference genome, the clean reads were *de novo*-spliced to obtain the reference sequence for subsequent analysis. The clean reads were spliced using the Trinity method (Sammeth et al., 2008; Grabherr et al., 2013), and the longest transcript of each gene was selected as the unigene. Trinity combines the three independent software modules of Inchworm, Chrysalis, and Butterfly to process and splice a large amount of RNA-seq data.

Unigene functional annotation: The *R. hybrida* unigene sequences were searched against six major databases (including Swiss-prot database, GO database, and KEGG database) obtain gene function annotations. GO, or gene ontology is a structured standard biological annotation system whose information applies to each species and can classify gene function in international standards. In this experiment, the distribution of all the spliced Unigene in GO was analyzed. After the differential genes were screened, the distribution of differential genes in GO was also analyzed, to analyze the function of differential genes before and after low-temperature treatment. KEGG is the primary public database for the analysis of metabolic pathways and gene function in cells. The functions and pathways associated with DEGs were determined by KEGG pathway analysis.

Gene transcript level analysis: To analyze the gene expression levels in samples before and after the freezing treatment, the sequence reads were mapped against the reference sequence using Bowtie software, and htseq-count (v0.6.0) (Robinson et al., 2010) was used to estimate each transcript’s readcount value. The gene expression level is expressed as the FPKM value, calculated as follows:


$$\text{FPKM}=\frac{\text{cDNA Fragments}}{\text{Mapped Fragments }\left(\text{millions}\right)\times \text{Transcript Length }\left(\text{kb}\right)}$$

where cDNA fragments represents the number of fragments obtained by paired-end sequencing aligned to a target transcript; mapped fragments (millions) is the total number of fragments aligned to all transcripts; and transcript length (kb) is the length of the transcript.

This experiment had three biological replicates, and the final expression value was the average of the three replicates.

Detection of DEGs from transcriptome data: The readcount data obtained in the gene expression level analysis were analyzed using the quantile standardization method with DESeq software. The threshold values were *p*-value<0.05 and FDR<0.05. Significant DEGs were defined as those with a -fold change of ≥2 (*p-*value<0.1, FDR<0.1) and common DEGs were defined as those with a -fold change of ≥1.5 between the two compared groups.

GO and KEGG analyses: We used BiNGO (v2.44) software (Maere et al., 2005) to perform GO enrichment analysis of the significant DEGs. This involved calculating the number of enriched genes per GO term in the overall background, and then applying a hypergeometric test to screen the GO terms that are significantly enriched with significant DEGs.

We used KOBAS 2.0 software (Sammeth, 2009) to detect pathways significantly enriched with DEGs in *R. hybrida* between before and after treatment at −15°C. According to the known pathways in KEGG, the hypergeometric test method to detect those significantly enriched with DEGs.

#### 2.2.5 qPCR verification of DEGs detected from transcriptome sequencing data

To verify the accuracy of the transcriptome data, 10 unigenes related to the low-temperature response, as detected based on the results of transcriptome data and GO/KEGG analyses (**Supplemental Table 9**), were selected for qRT-PCR analysis. The data from the two analyses were compared in a correlation analysis. For the qRT-PCR method, see section 2.3 below.

### 2.3 Transcript profiles of cold tolerance-related genes in *R. hybrida*

Leaves of *R. hybrida* collected at different time points during the −15°C freezing treatment were used for the screening of internal reference genes and the analysis of DEG profile patterns.

#### 2.3.1 cDNA synthesis

The cDNA was synthesized using a Promega Reverse Transcription System kit (Promega, Madison, WI, USA) according to the manufacturer’s instructions.

#### 2.3.2 Semi-quantitative RT-PCR

We selected three candidate reference genes for *R. hybrida*; *UBI1* and *ACT4*, which were used in a previous study on gene expression in *Rosa* (Meng et al., 2013), and *GAPDH*, which was used in a study on changes in gene expression of *Rosa viciifolia* under cold stress (Liu, 2017).

The internal reference genes were screened to determine which one was most suitable for our analyses using semi-quantitative RT-PCR. The 25-µL reaction mixture included 1 µL each of upstream and downstream primers (10 µM), and 2 × Novartis Taq Plus Master Mix (12.5 µL), 1 µL cDNA, and 9.5 µL ddH_2_O. The analyses were conducted using a Bio-Rad T100 gradient PCR instrument, with the following thermal cycling conditions: 94°C for 4 min for pre-denaturation, followed by 30 cycles of 94°C for 30 s, 59°C for 30 s, and 72°C for 30 s, and then final extension at 72°C for 5 min. The sequences of the three internal reference primers tested are shown in **Supplemental Table 1**. The amplification reaction products were detected by 1% agarose gel electrophoresis.

#### 2.3.3 Real-time quantitative PCR

The internal reference gene for qRT-PCR was *UBI1*, which was amplified using the primers described by Meng et al. (2013). Other gene primers were designed using Primer Premier 5 software, and the sequences are shown in **Supplemental Table 1**.

The qRT-PCR analyses were conducted using TB Green TM Fast qPCR Mix (#RR430A) on a CFX96 Real Time PCR Detection System. The thermal cycling conditions were as follows: pre-denaturation for 1 min at 95°C, followed by 40 cycles of 95°C for 5 s and 60°C for 30 s. Melting curve analysis was conducted to confirm the specificity of the primers. The relative gene transcript levels were calculated as follows:


$${Relative gene expression=2}^{-}{\;}^{\Delta \Delta \text{Cq}}$$

where△Cq refers to the difference between the Cq values of the target gene and the reference gene, and △△Cq refers to the difference between the△Cq values of the treatment and the control.

## 3 Results

### 3.1 Cold tolerance of nine *Rosa* materials

#### 3.1.1 Changes in REC of *Rosa* materials under low temperature

**Figure 1:**
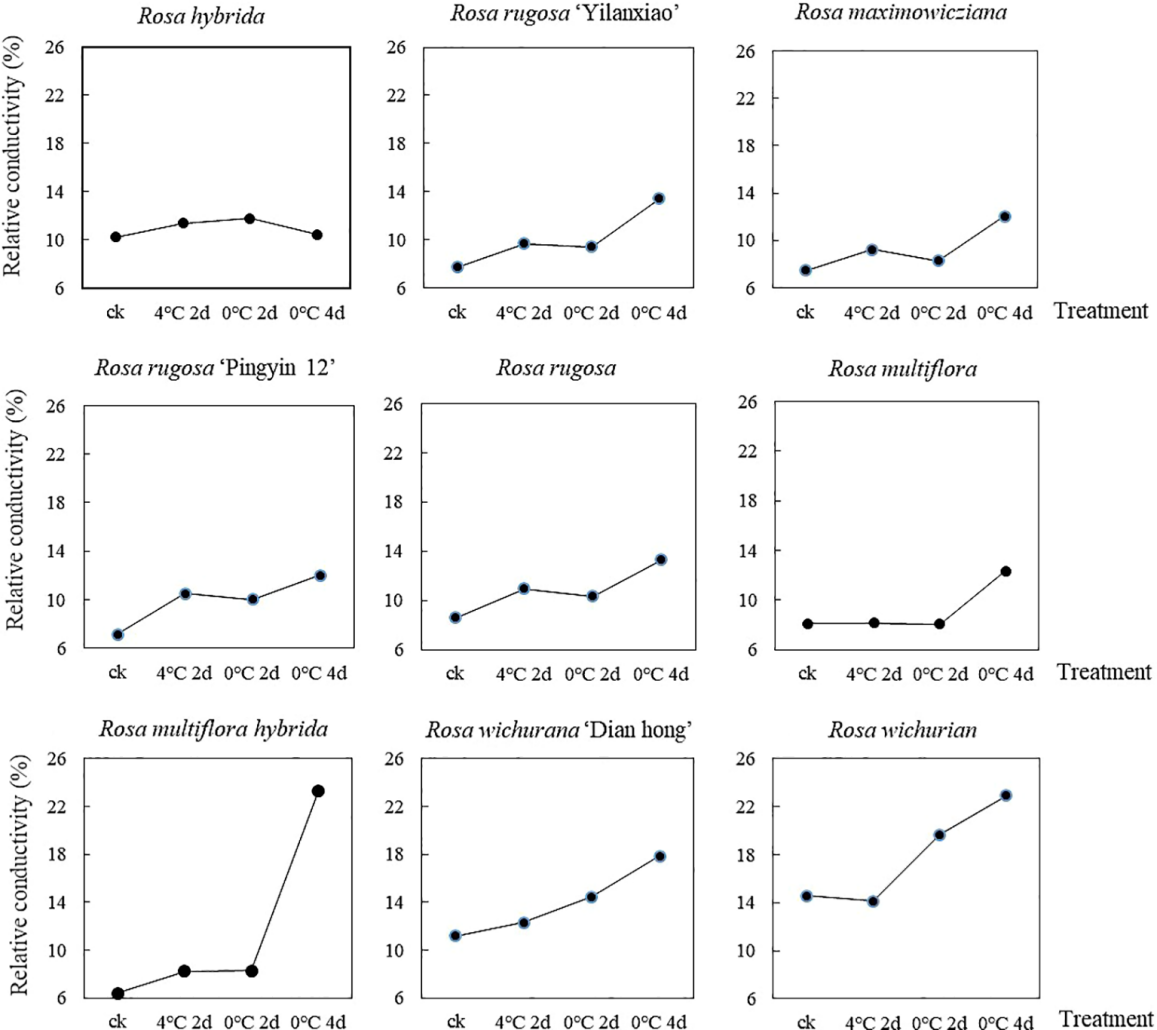
Changes in REC of nine *Rosa* materials under a low-temperature treatment.

The REC of the nine *Rosa* materials increased as the duration of the low-temperature treatment increased, but the extent of the increase differed among the materials (**Figure 1**). The overall REC increased by 16.1% after treatment at 4°C for 2 d, by 23.2% after treatment at 0°C for 2 d, and by 69.1% after treatment at 0°C for 4 d. For *R. multiflora*, the REC was almost unchanged after treatment at 4°C for 2 d and 0°C for 2, but reached the maximum value after treatment at 0°C for 4 d. During the low-temperature treatment, the REC of *R. hybrida* increased first and then decreased. The REC of the other materials did not increase significantly after treatment at 4°C for 2 d and 0°C for 2 d, but increased and reached the maximum value after treatment at 0°C for 4 d. Among the nine materials, *R. multiflora hybrida* showed the largest increase in REC after treatment at 0°C for 4 d (to 3.65 times that of the control group). These results showed that low-temperature treatment at 4°C for 2 d and 0°C for 2 d caused little damage to cell membranes. However, when the treatment time at 0°C increased to 4 d, there was substantially more damage to cell membranes. Thus, the longer the treatment time at low temperature, the greater the damage to the cell membrane system. This resulted in increased permeability and the release of electrolytes, which caused the REC value to increase.

To eliminate the influence of initial damage among the different materials, the membership function method was used to process the measured REC values of the nine materials. The larger the membership function value, the stronger the cold tolerance of the material. The nine materials were ranked, from highest membership function value to lowest, as follows: *R. rugosa* ‘Pingyin 12’ > *R. hybrida* > *R. rugosa* > *Rosa wichurian* > *Rosa wichurana* ‘Dian hong’ > *R. rugosa* ‘Yilanxiao’> *Rosa maximowicziana* > *Rosa multiflora hybrida* > *Rosa multiflora.*

#### 3.1.2 Changes in plant morphology under cold treatment

**Figure 2:**
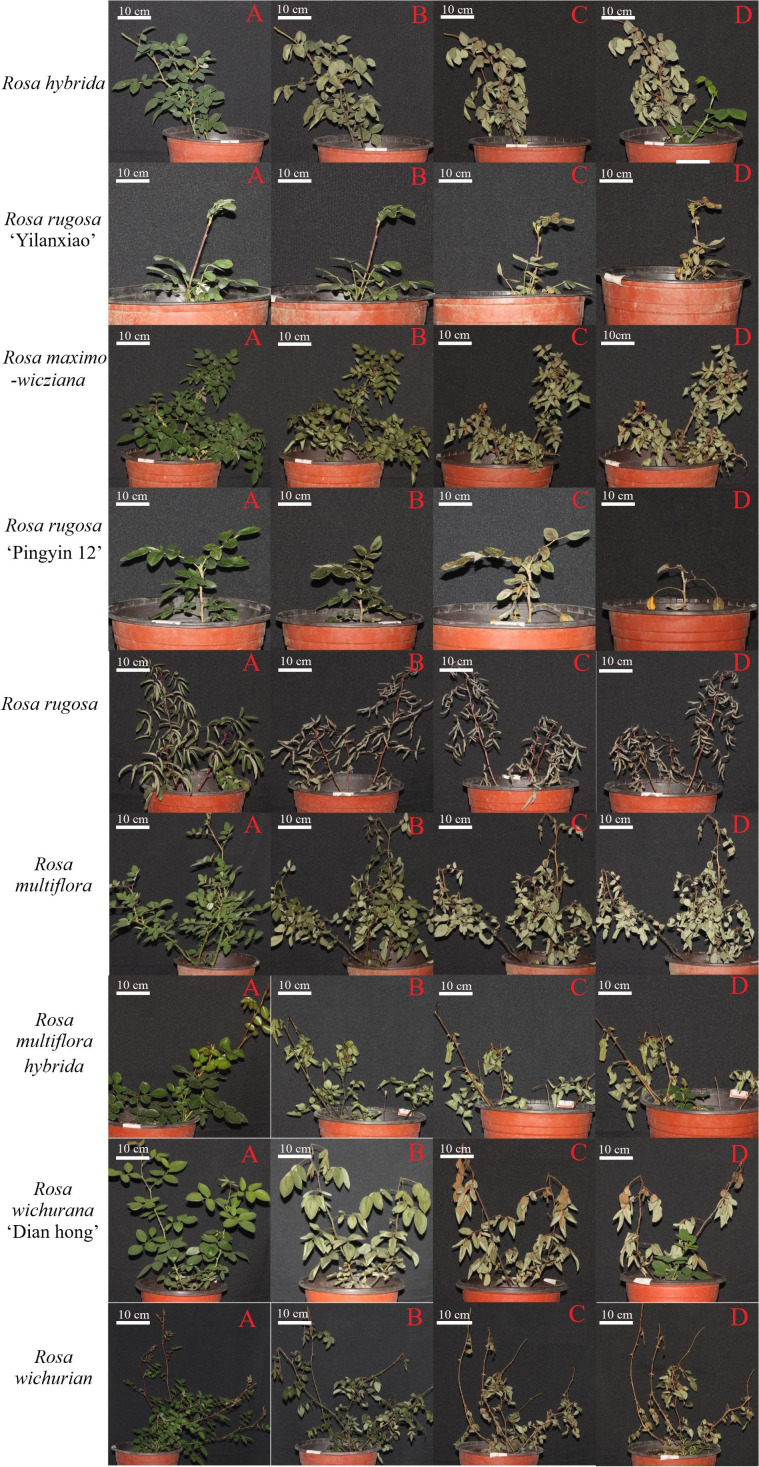
Morphology of *Rosa* materials before cold treatment **(A)** and at 1 d **(B)**, 15 d **(C)**, and 30 d **(D)** of recovery after the low-temperature treatment.

Leaves are the first organ to show symptoms of low-temperature stress. **Figure 2** shows the morphology of the *Rosa* materials before the low-temperature treatment and during 30 d of recovery afterwards. During recovery, the leaves dried, shriveled, and lost their green color. Some became yellow. The branches changed in color and gradually became dehydrated.

The plant morphology changed by day 1 of recovery, with the leaves becoming wilted, drooped, and less glossy, and some branches showing color changes. White spots appeared on the leaves of *R. hybrida* and *R. rugosa* ‘Pingyin 12’, and the leaves of *R. rugosa* turned gray. Among all the materials, *R. rugosa* ‘Yilanxiao’ showed the smallest changes in leaf morphology. The branches of *R. rugosa* ‘Yilanxiao’ and *R. rugosa* remained purple, and branches of *R. rugosa* ‘Pingyin 12’ did not change color either. The stems of the other plants turned purple or black to varying degrees. The branches of *R. wichurian* were slender and widely spread. After the low-temperature treatment, the branches changed from purple to dark green, especially the lower branches, and their tips wilted.

At 15 d of recovery, the discoloration, yellowing, and dullness of the leaves had become more severe. The leaves of *R. rugosa* ‘Yilanxiao’, *R. rugosa* ‘Pingyin 12’, and *R. wichurana* ‘Dian hong’ were yellow, with obvious discoloration around the veins. The shoots of *R. multiflora* and *R. wichurian* were black and yellow. The branches on the upper third of *R. hybrida* and *R. wichurana* ‘Dian hong’ plants were yellow, but these two materials had produced new shoots at the base.

At 30 d of recovery, the branches of nine *Rosa* materials had changed color. There were multiple buds at the leaf positions on the shoots of *R. rugosa* ‘Pingyin 12’ and *R. rugosa*, but they were clustered together, indicating that the branches had not grown. Most of the branches on the other seven materials were still actively growing. As shown in **Figure 2**, new branches were present at the base of *R. hybrida*, *R. maximowicziana*, *R. multiflora hybrida*, *R. wichurana* ‘Dian hong’, and *R. wichurian* Both *R. hybrida* and *Rosa wichurana* ‘Dian hong’ had formed multiple compound leaves and showed the strongest recovery potential. *R. rugosa* ‘Yilanxiao’ and *R. multiflora* did not show form any new buds and did not survive.

On the basis of the results of the low-temperature treatments, *R. hybrida*, *R. rugosa* ‘Pingyin 12’, and *R. rugosa* were identified as the most cold-tolerant materials, while *R. rugosa* ‘Yilanxiao’ and *R. multiflora* were identified as the least cold tolerant. *R. hybrida* was chosen for transcriptome analysis because it showed a strong recovery potential after the low-temperature treatment.

### 3.2 Detection of DEGs based on transcriptome data

#### 3.2.1 Phenotypic changes of *R. hybrida* plants before and after freezing treatment

**Figure 3:**
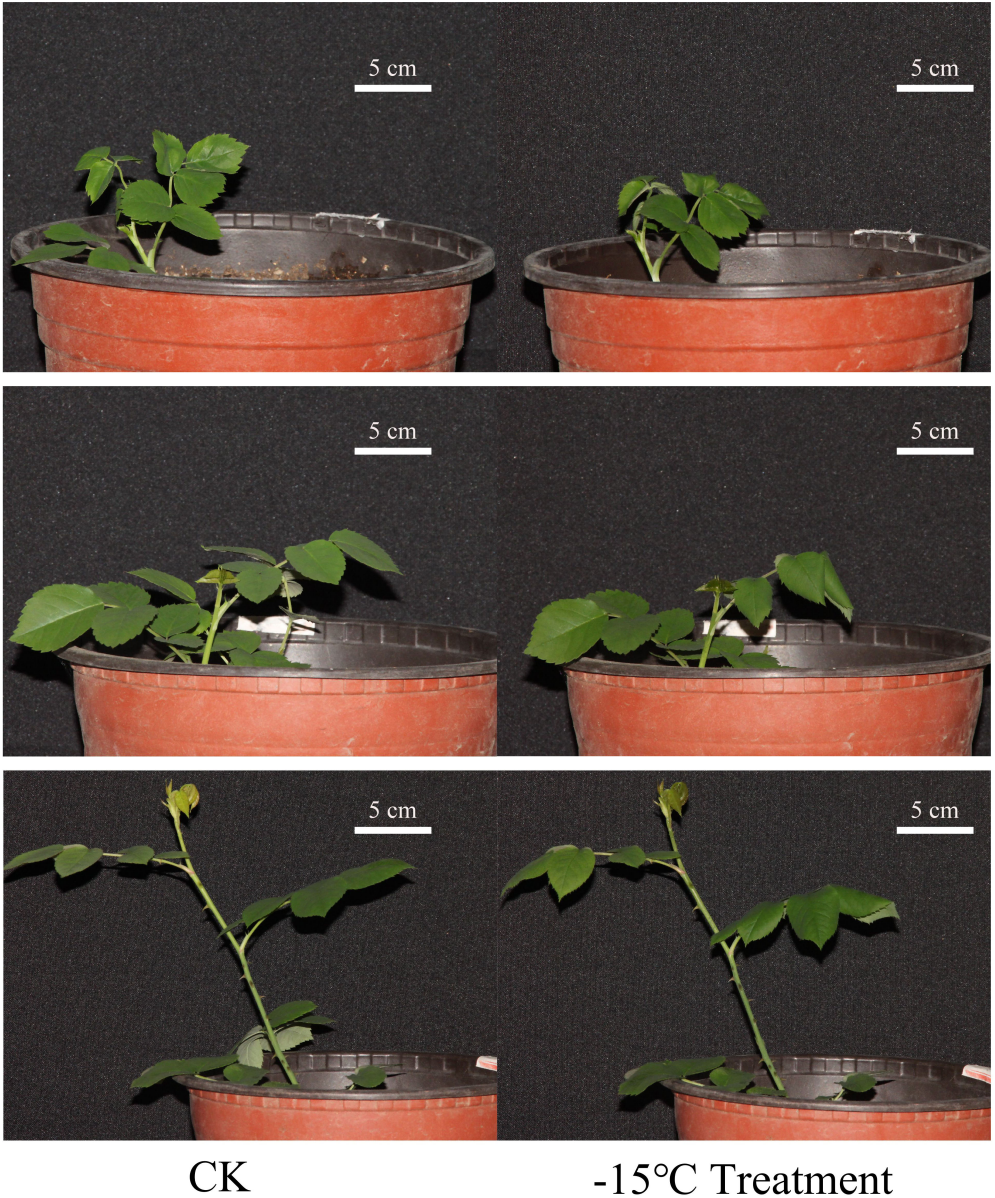
Morphological changes in *R. hybrida* plants after 10 min treatment at −15°C.

To explore the molecular mechanism of cold resistance of *R. hybrida*, we selected *R. hybrida* plants after 30 d of recovery from the low-temperature treatment, and then subjected them to a freezing treatment (−15°C for 10 min). As shown in **Figure 3**, the leaflets of the three *R. hybrida* plants curled and drooped during the freezing treatment.

#### 3.2.2 RNA sample quality

The six RNA samples produced clear bands, indicative of high integrity of the samples (**Supplemental Figure 1**). Analyses of the A260/A280 values (1.9≤A260/280 ≤ 2.1) (**Supplemental Table 3**) indicated that the samples were of sufficient quality for library construction.

As shown in **Supplemental Figure 2**, the cDNA library amplification curve had a single peak, with no contaminants or primer dimers. The initial concentration of the six samples was higher than 15.68 nM (**Supplemental Table 4**), so they met the requirements of the HiSeq 2500 sequencing platform. The size of the inserted fragments was suitable for further analysis. Thus, the quality of the constructed transcriptome library was sufficient for further processing steps.

#### 3.2.3 Sequencing quality and transcript splicing

The six RNA samples from leaves of *R. hybrida* were subjected to RNA-Seq analysis. The total number of bases was 28.552 GB (at least 4.304 GB per sample). The unigene data from the six samples were combined to establish a *R. hybrida* unigene library. The Q20 base percentage was >95.63%, the Q30 base percentage was >89.97%, the GC content was >46.89%, and the average quality was higher than 37.71 (see **Supplemental Table 5**), indicating that the sequencing quality was sufficient for further analyses.

**Figure 4:**
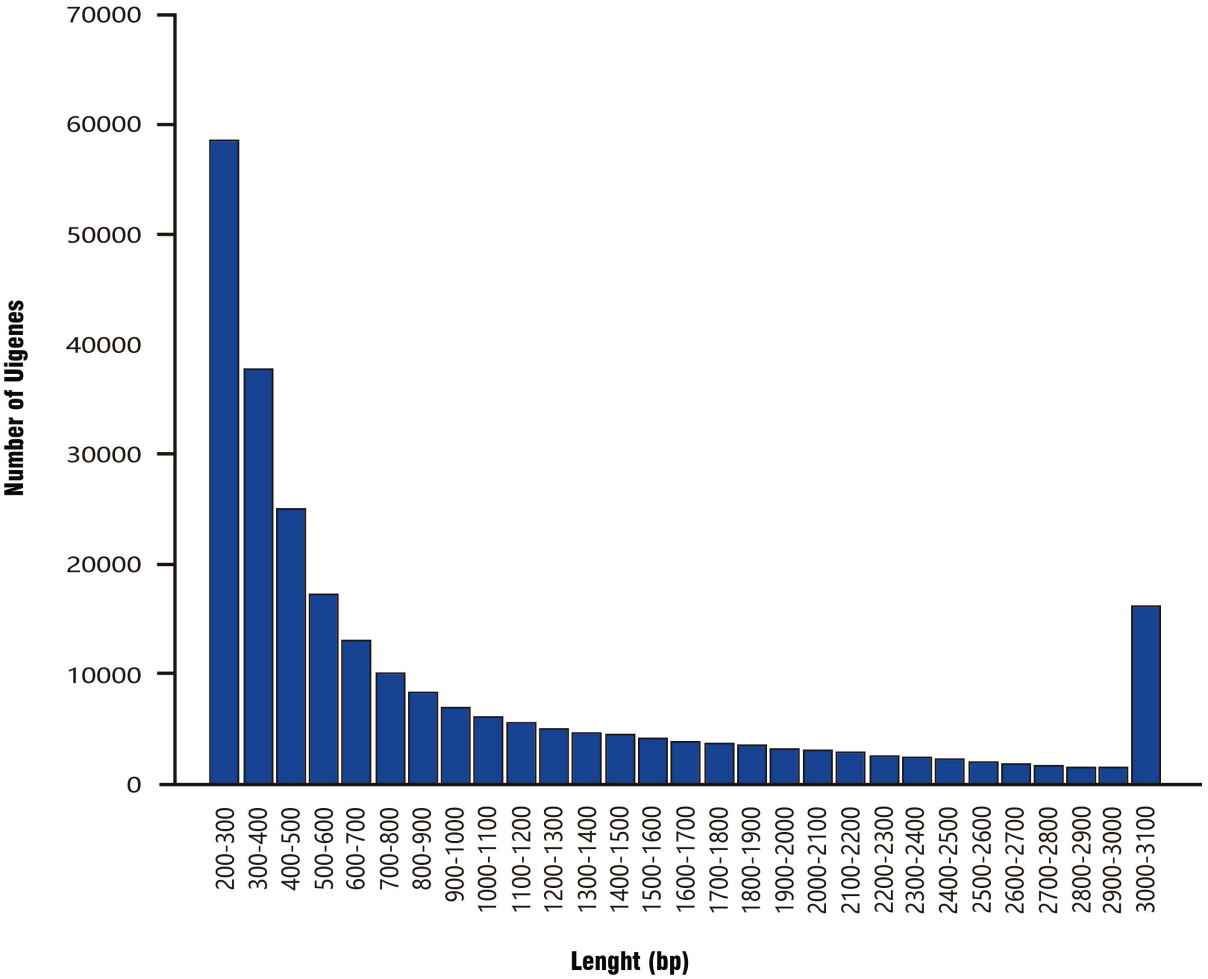
Distribution of unigene length in the library.

After splicing using Trinity software, the total number of bases was 261.64 MB, and 258,055 unigenes were obtained. The unigene length distribution curve was smooth (**Figure 4**). The average sequence length was 1013.9 bp, with the longest sequence being 27,201 bp. There were 76,022 unigenes longer than 2000 bp, and they accounted for 29.46% of the total unigene library. Further statistical information is provided in **Table 1**. The N50 (the length of the shortest contig for which longer and equal-length contigs cover at least 50% of the assembly) was 1,844. The larger the N50 value, the longer fragments are assembled and the better the assembly. This high N50 value confirmed the high integrity of the assembly.

**Table 1:** Unigene length frequency distribution table.

Transcription length distribution	200–500 bp	500–1000 bp	1000–2000 bp	>2000 bp	Total
**Unigene quantity**	121081	55134	43801	76022	258055

#### 3.2.4 Unigene annotation

To explore the significance of differences in gene expression in *R. hybrida* under short-term freezing conditions, unigenes were annotated using GO, KEGG, and six other databases.

**Figure 5:**
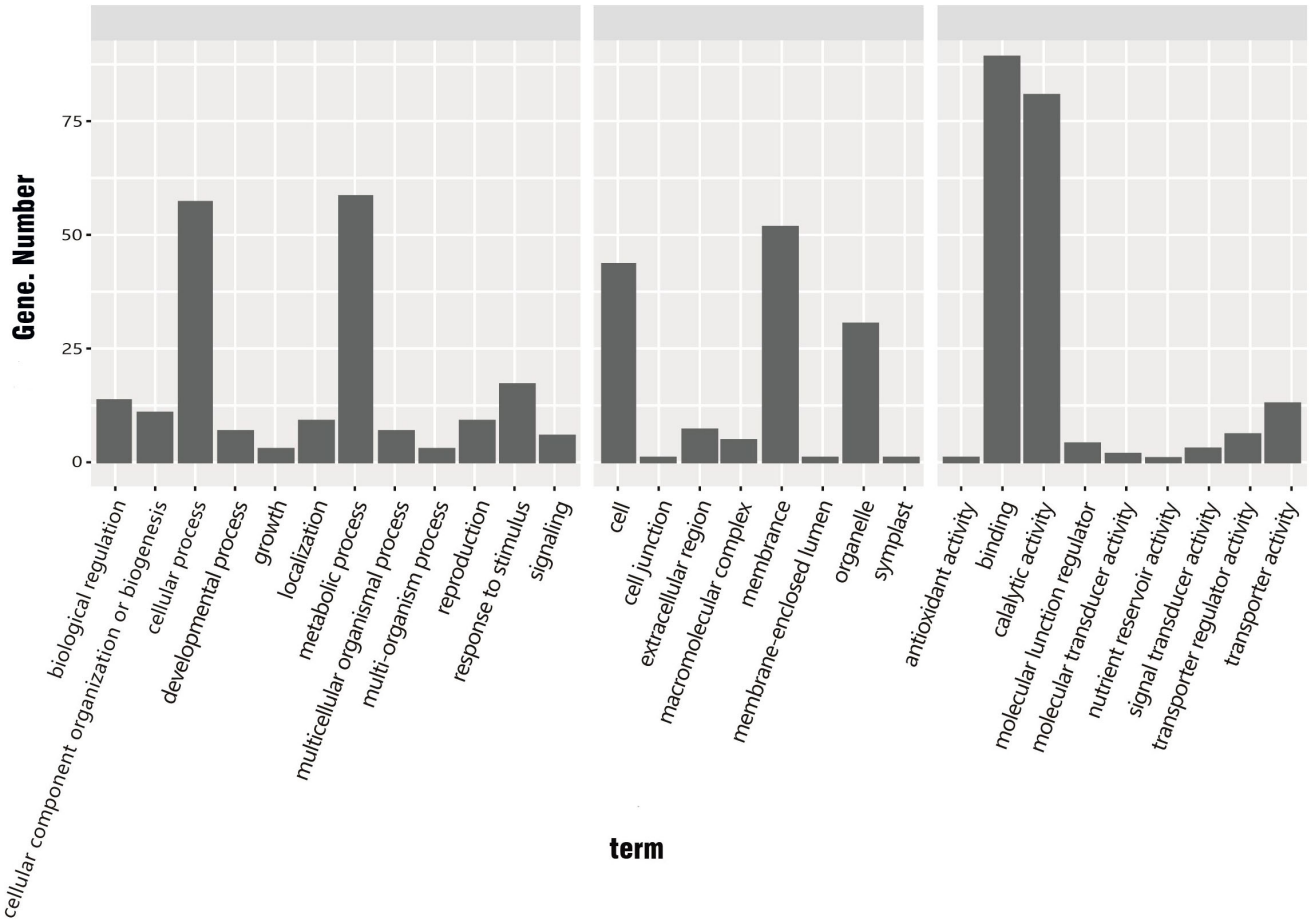
GO classification of common DEGs in *R. hybrida*, i.e., genes showing ≥1.5 log2-fold change between cold treatment and control. (BP, biological process; CC, cellular component; MF, molecular function).

The 534 common DEGs were searched against the GO database. The unigenes with GO annotations were distributed in 29 subcategories of three major categories: Biological process (BP), Cellular component (CC), and Molecular function (MF). The DEGs were enriched in 12 subcategories of the BP category, eight subcategories of CC, and nine subcategories of MF. Among these subcategories, those enriched with large numbers of DEGs were protein binding (88 DEGs), catalytic activity (80 DEGs), metabolic process (58 DEGs), cellular process (57 DEGs), membrane (51 DEGs), cell (43 DEGs), and organelle (30 DEGs) (**Figure 5**).

Many genes contribute to normal biological functions in organisms. Pathway enrichment analysis can determine the most important biochemical metabolic pathways and signal transduction pathways involving the target gene, and this can shed light on the molecular mechanisms underlying physiological responses. In this experiment, 2192 genes were compared with the KEGG database. The common DEGs participated in 367 KEGG pathways. The pathways most enriched with the common DEGs were RNA transport, MAPK signaling, and starch and sucrose metabolism.

#### 3.2.5 Transcriptome SNP analysis

An SNP is a change in a single nucleotide base in the genome. In simple terms, it is the same among different individuals. There is only one difference in the fact that most of the base sequences in the nucleotide sequence of a chromosome or the same site are the same. There are many SNPs in the genome, and multiple SNPs may be present on the same chromosome. SNP markers can be developed by screening for SNP sites closely linked to biological traits. Theoretically, each SNP site can have four different variants. Single nucleotide variants with a mutation frequency greater than 1% are called SNPs.

We used SAMtools/BCF tools to compare the sample genome with the reference genome, and then performed sorting, deduplication, and other processing steps. Finally, we used the mutation detection software SAMtools for SNP calling and filtering of the final SNP dataset. We detected about 800,000 SNPs in each of the six samples (average value, 824,829.5; lowest value, 776,355) indicating that differences among the six samples were small, and the sequencing and splicing results were accurate (**Supplemental Table 6**).

#### 3.2.6 Gene transcript level analysis

**Figure 6:**
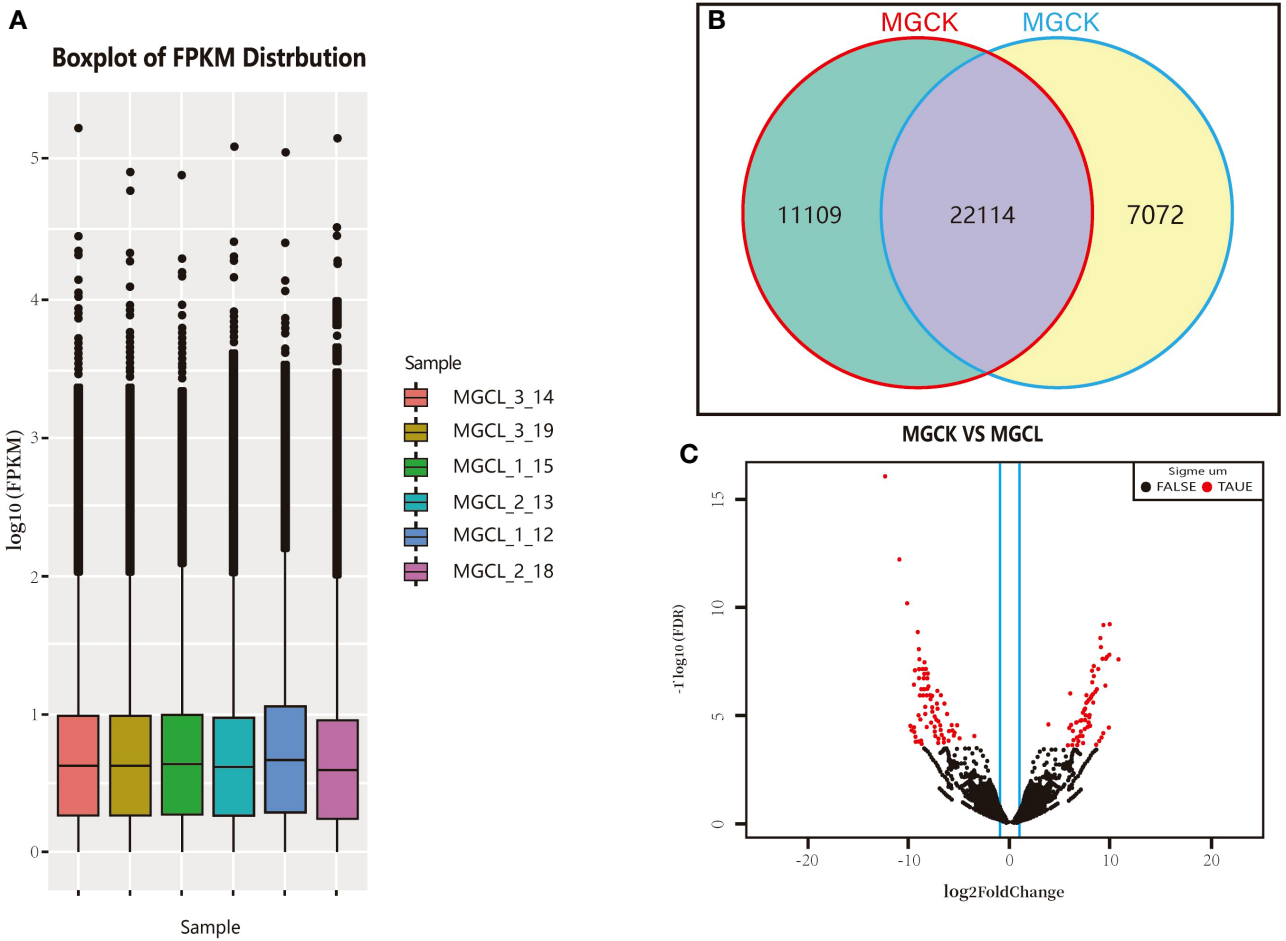
Gene transcript profiles in experimental materials and cluster volcanic plot of significant DEGs. **(A)** Log FPKM values. **(B)** Number of genes expressed in control (MGCK, no cold treatment) and under cold treatment (MGCL). **(C)** Cluster volcano plot of significant DEGs, red and black dots indicate genes showing significant and common DEGs, respectively, between control and cold treatment.

The FPKM value was obtained for each unigene as described above. The FPKM values spanned 10^0^–10^5^ orders of magnitude. The limits, median, and upper and lower quartiles of FPKM values, as well as deviations, were similar between the control (pre-freezing) and freezing treatment (−15°C for 10 min). The overall gene expression level after the freezing treatment was slightly higher than that before (**Figure 6A**). As shown in **Figure 6B**, 22,114 genes were expressed both before and after the low-temperature treatment, 11,109 genes were expressed only before the freezing treatment, and 7,072 genes were expressed only after the freezing treatment.

#### 3.2.7 Screening to detect DEGs after freezing treatment

We used DESeq software to process the readcount values representing the gene expression levels, and used the quantile standardization method to screen significant DEGs between before and after the freezing treatment (genes with *p-*value<0.05, FDR<0.05, and |log2(fold change)|≥2T). The log2(fold change) indicates the size of the difference in gene transcript levels between two groups of samples, and the FDR is the correction value of the statistically significant *p*-value. As shown in **Figure 6C**, there were more significant DEGs than common DEGs. The distribution of the significant DEGs was more discrete than that of common DEGs.

We detected a total of 204 significant DEGs between before and after the cold treatment, accounting for 0.079% of all unigenes. Of them, 88 were significantly up-regulated and 116 were significantly down-regulated.

#### 3.2.8 GO classification and enrichment analyses of significant DEGs

**Figure 7:**
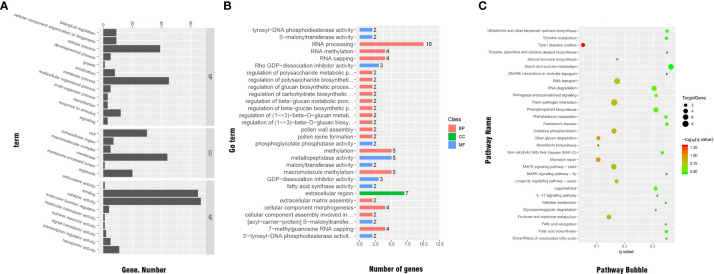
**(A)** GO classifications of significant DEGs. **(B)** Distribution of GO functional categories. **(C)** Functional annotations of significant DEGs by KEGG analysis.

We conducted GO classification and enrichment analyses to explore the functions of the significant DEGs. In total, 137 significant DEGs were subjected to GO classification analysis. These DEGs were distributed in 27 subcategories within the main three categories of BP (12 subcategories), MF (9 subcategories), and CC (6 subcategories). Within the MF category, the subcategories most enriched with DEGs were ‘catalytic activity’ (67 DEGs), ‘protein binding’ (65 DEGs), ‘metabolic process’ (45 DEGs), ‘cell membrane’ (44 DEGs), ‘cellular process’ (39 DEGs), ‘cell’ (30 DEGs), and ‘organelle’ (20 DEGs). We note that there may be duplicate unigenes in each category (**Figure 7A**).

The results of GO enrichment for significant DEGs are shown in **Figure 7B**. In total, 47 significant DEGs were enriched in various categories and subcategories, with the BP category having the largest number of DEGs and the CC category having the fewest DEGs. Ten of the DEGs were enriched in the ‘RNA processing’ subcategory. Other subcategories enriched with significant DEGs under cold stress were ‘regulation of cellular carbohydrate metabolic process,’ ‘regulation of beta-glucan biosynthetic process,’ ‘regulation of beta-glucan metabolic process,’ and ‘malonyltransferase activity,’ which is related to the electron transfer chain. Therefore, these processes and pathways may be related to cold resistance in *Rosa.*

#### 3.2.9 KEGG pathway enrichment analysis of significant DEGs

The KEGG pathway enrichment analysis showed that, across the genome of the *R. hybrida*, 45 significant DEGs were enriched in 57 pathways in five categories and 16 subcategories. The KEGG classification results are shown in **Supplemental Table 7**. The pathways most enriched with DEGs were ‘carbohydrate metabolism’ (in the metabolism category) and ‘signal transduction’ (in the environmental information processing category).

The pathways most enriched with DEGs were ‘RNA transport’ (6 DEGs), ‘starch and sucrose metabolism’ (5 DEGs), ‘phenylpropanoid biosynthesis’(5 DEGs), ‘MAPK signaling pathway-plant’ (5 DEGs), ‘mismatch repair’ (4 DEGs), oxidative phosphorylation (4 DEGs), and ‘fructose and mannose metabolism’ (4 DEGs) (**Figure 7C**).

Some of the pathways enriched with DEGs, including ‘fatty acid elongation,’ ‘biosynthesis of unsaturated fatty acids,’ and ‘aminoacyl-tRNA biosynthesis’ were related to the changes in unsaturated fatty acid content in biofilms and proline metabolism during the response to low-temperature stress. The ‘oxidative phosphorylation,’ ‘glycolysis/gluconeogenesis,’ and ‘starch and sucrose metabolism’ pathways were related to intracellular metabolism and the accumulation of protective substances against cold-induced damage. The ‘plant hormone signal transduction’ pathway was related to signal transduction and hormonal regulation under low-temperature stress. The pathways significantly enriched with DEGs are shown in **Supplemental Table 8**.

#### 3.2.10 qPCR verification of DEGs identified from transcriptome data

**Figure 8:**
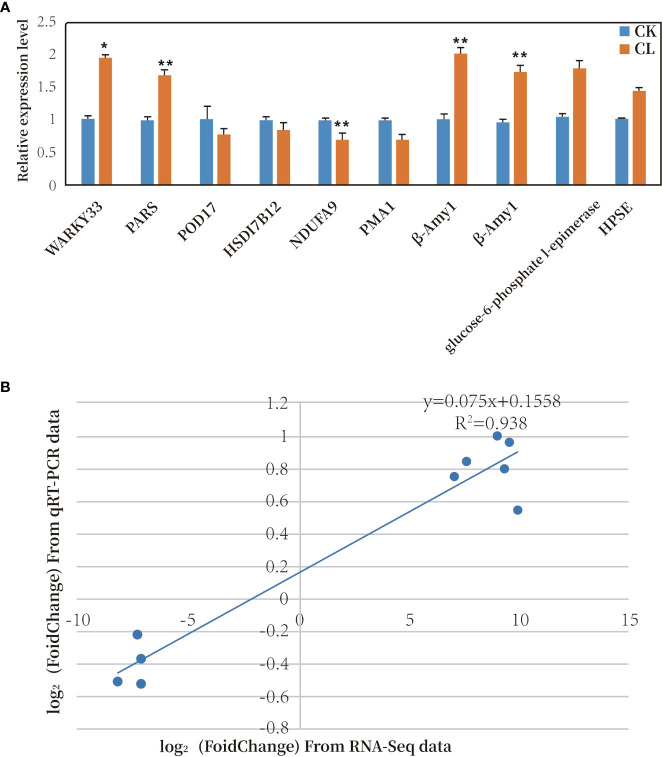
**(A)** Relative transcript levels of 10 DEGs as determined by qRT-PCR. * represents significant difference at *P*< 0.05 compared with control; ** represents significant difference at *P*< 0.01 compared with control. **(B)** Correlation analysis of results of RNA-Seq and qRT-PCR analyses of 10 DEGs. (CK, MGCK, no cold treatment; CL: MGCL, under cold treatment).

Ten genes were selected for qRT-PCR analysis. Six of the 10 genes were up-regulated and four were down-regulated under freezing stress (**Figure 8A**). The trends in gene transcription detected in the qPCR analyses were the same as those detected from the transcriptome data (see **Supplemental Table 9**). A correlation analysis was conducted between the transcriptome sequencing log2 (Fold Change) values and the qRT-PCR relative expression log2 (Fold Change) values. The two sets of data were positively correlated, with a correlation coefficient (R^2^) of 0.938 (**Figure 8B**). These results confirmed that the transcriptome data were accurate and reliable.

### 3.3 Trends in transcription of cold-tolerance-related genes in *R. hybrida*

#### 3.3.1 Internal reference gene screening

We conducted semi-quantitative RT-PCR analyses to screen for an appropriate internal reference gene. The product amplified with *GAPDH* primers had two bands, indicative of poor primer specificity. Single bands were produced using the *UBI1* and *ACT4* primers, indicating that they had better specificity (**Supplemental Figure 3**). Thus, *UBI1* and *ACT4* were selected as potential internal reference genes for qRT-PCR.

The *UBI1* and *ACT4* genes were subjected to qRT-PCR, and the cq values obtained were processed and analyzed using NormFinder and BestKeeper software. The results showed that *UBI1* expression was more stable than *ACT4* expression (**Table 2** and **Supplemental Table 10**). Therefore, *UBI1* was selected as the reference gene in this study.

**Table 2:** Expression stability of *UBI1* and *ACT4* analyzed by NormFinder.

Gene	stability	Sort
*UBI1*	0.018	1
*ACT4*	0.028	2

#### 3.3.2 Transcript profiles of cold-tolerance-related genes in *R. hybrida* at −15°C

After analyzing the results of transcriptome sequencing, we selected four genes related to cold tolerance for verification of their transcriptional profiles at different times points under −15°C treatment by qRT-PCR.

Note: * indicates significant difference (*P*< 0.05) compared with 0 h; ** indicates significant difference (*P*< 0.01) compared with 0 h.

**Figure 9:**
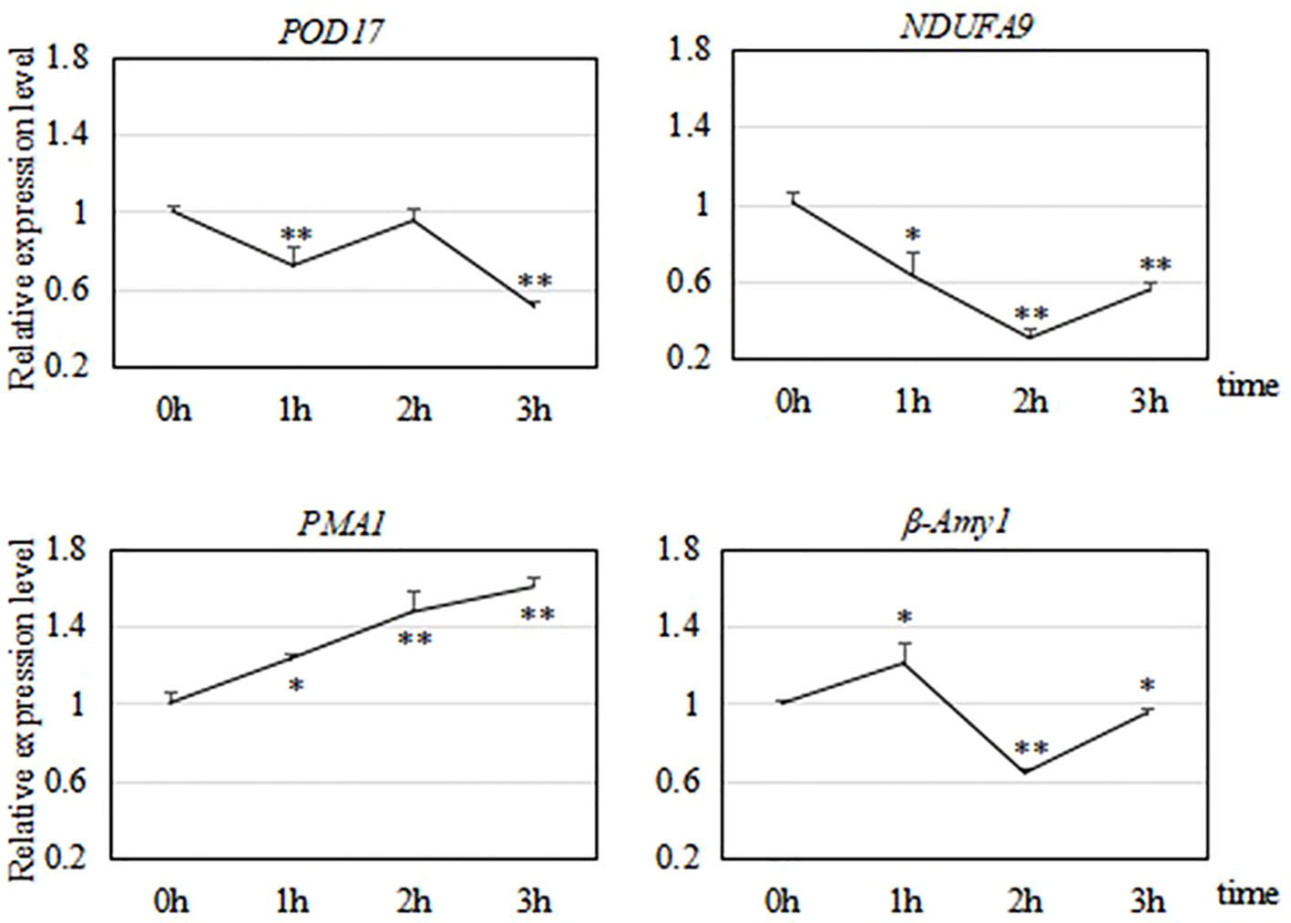
Relative transcript levels of four genes during a 3-h freezing (−15°C) treatment. * indicates significant difference (*P* < 0.05) compared with 0 h; ** indicates significant difference (*P* < 0.01) compared with 0 h.

As shown in **Figure 9**, we analyzed the transcript levels of four genes related to the cold response of *R. hybrida*. One of them encoded a POD, which participates in the enzymatic protection system in plants. During the 3-h freezing treatment, *POD17* was significantly down-regulated at 1 h, up-regulated at 2 h, and then down-regulated to the lowest level at 3 h. We speculated that the strongest response at 2 h would result in the production of POD to increase the capacity of the enzymatic protection system.

*NDUFA9* encodes a subunit of NADH dehydrogenase, which catalyzes the transfer of electrons from NADH to coenzyme Q. This is a crucial enzyme for oxidative phosphorylation in mitochondria. During the 3-h freezing treatment, *NDUFA9* was down-regulated during the first two h, and then up-regulated by 3 h.

*PMA1* encodes an ATPase. During the 3-h freezing treatment, *PMA1* was continuously and significantly up-regulated, with highly significant increased transcript levels at 2 h and 3 h (to 1.6 times that in the control).

The expression of *β-Amy1* increased first, then decreased, and then increased during the freezing treatment. It was significantly up-regulated at 1 h (to 1.2 times that in the control), decreased to its lowest level at 2 h, and then was up-regulated at 3 h. This fluctuation in the transcript level of *β-Amy1* in *R. hybrida* suggested that the abundance of amylase was regulated to control the soluble sugar (maltose) content in the cells.

## 4 Discussion

### 4.1 Cold tolerance of *Rosa* materials

Plant cells sense low temperature through receptors on the membrane. The transmission of signals related to the low temperature response results in damage to the structure and function of the cell membrane system, changes in membrane permeability, and a large amount of electrolytes leaking from the membrane. This can be detected as an increase in REC. In cold-tolerant plants, the cell membrane permeability will change less and gradually recover. However, in cold-sensitive plants, the cells are more severely damaged and membrane permeability is greatly increased. If the cells cannot recover, this damage can result in plant death. In this study, we found that the REC values of two materials, *R. hybrida* and *R. rugosa* ‘Pingyin 12’, were lower than those of materials after cold treatment at 4°C for 2 d and 0°C for 4 d. The REC values of *R. hybrida* were particularly low, indicating that its membranes were not severely damaged by the cold treatment, and were able to recover afterwards. On the basis of this analysis, we concluded that *R. hybrida* and *R. rugosa* ‘Pingyin 12’ are cold-resistant *Rosa* materials.

In this study, whole plants in the *Rosa* genus were subjected to a low-temperature treatment to evaluate their cold tolerance. In other studies, such as those of Ren (2013) and Ma and Chen (1991), the cold tolerance of *Rosa* materials was assessed using isolated leaves and branches. One study assessed cold tolerance using dormant branches of *Rosa* plants, and measured changes in physiological indicators after a low-temperature treatment. Although that method is economical, convenient, and saves experimental space, the mechanical damage caused by cutting will affect the accuracy of the experimental results. In addition, because most of the branches and leaves are used, there is no way to observe the morphological changes to living plants after a low-temperature treatment. In this study, the cold tolerance of *Rosa* materials was evaluated by subjecting whole plants to a low-temperature treatment, and then assessing various physiological indicators and recovery. Compared with using cuttings, using whole plants provides a more accurate picture of how they respond to, and recover from, low-temperature stress.

Plant leaves produce carbohydrates through photosynthesis and then transport them to the branches and roots. The carbohydrates produced by photosynthesis are related to cold resistance (Liu, 2017). The sucrose produced in the leaves not only functions as a nutrient for transport to other organs *via* the phloem, but also as a response signal to various stresses. Price et al. (2004) found that a large number of stress-responsive genes, such as genes related to carbohydrate metabolism, signal transduction, and metabolite transport, are induced by glucose. Most of these genes are related to the cold resistance of plants, indicating that glucose in plant leaves plays an important role in signal transduction to establish cold resistance. Liu (2017) measured several physiological indicators in the branches and leaves of *Rosa sylvestris* and *C. odorifera* after a low-temperature treatment. Although the dynamic changes under low temperature were consistent for most indicators, they were inconsistent for soluble sugar content. In this study, we measured the REC of leaves after a low-temperature treatment and growth recovery after a −15°C treatment. We found that the results of growth recovery were consistent with the REC results. Therefore, determining the REC of leaves after a low-temperature treatment is a reliable method to assess the cold tolerance of plants.

According to our results, the most cold-resistant materials in this study were *R. hybrida* from Henan, *R. rugosa* ‘Pingyin 12’, and *R. rugosa* from Shandong, while *R. multiflora* and *R. wichurian* from Yunnan showed relatively weak cold resistance. This led us to speculate that their cold tolerance was related to their long-term growth environment. The most cold-tolerant materials are mainly grown in Northern China, while the most cold-sensitive materials are mainly grown in Southern China. It was speculated that differences in the cold tolerance of *Rosa* plants were related to the adaptability of provenances to their long-term growth environment.

### 4.2 Detection of DEGs from transcriptome data

The molecular mechanism of cold tolerance in plants is complex. The physiological and biochemical changes in plants caused by low temperature are not regulated by a single gene. A gene can participate in several different pathways, and can be regulated by multiple upstream genes. Therefore, it is important to study the whole functional network. By analyzing the transcriptome data, we detected a large number of DEGs in *R. hybrida* under low-temperature stress. In further research, it will be important to explore the roles of these DEGs, and determine which ones encode key components in terms of cold resistance. In this study, we detected SNP markers for key genes related to cold resistance in *Rosa*. These may be useful for rapid estimates of the cold tolerance of various *Rosa* materials.

Transcription factors play an important role in the response to low-temperature stress. In this study, we detected significant up-regulation of the *WRKY33* transcription factor gene in the MAPK signaling pathway in *R. hybrida* under low-temperature stress. Many studies have demonstrated that this transcription factor is involved in resistance to stresses, including cold stress, in plants. (Jiang and Deyholos Michael, 2009). found that overexpression of *AtWRKY33* improved salt tolerance, and that ABA induced the expression of *OsWRKY08* and improved the osmotic stress resistance of transgenic *Arabidopsis* plants (Song et al., 2010). transferred soybean *WRKY21* into *Arabidopsis*, and found that it improved the cold tolerance of the transgenic plants. Another study demonstrated that *WRKY33* in alfalfa was up-regulated by low temperature (Feng et al., 2015). Further research is required to explore the roles of *WRKY33* in the cold response of *R. hybrida*, and to determine whether it can confer cold resistance.

The KEGG pathway enrichment analyses showed that POD is involved in three pathways simultaneously: ‘phenylpropanoid biosynthesis,’ ‘metabolic pathways,’ and ‘biosynthesis of secondary metabolites.’ Among all the pathways affected by the freezing treatment, ‘synthesis of phenylpropanol substances,’ was the most strongly affected. This pathway may be related to lignin synthesis. Lignin metabolism is related to plant cell differentiation, disease resistance, and fruit development (Yu et al., 2003). These results suggest that low temperature affects lignin synthesis in *Rosa*.

Liu (2017) conducted transcriptome analyses of *R. sylvestris*, and detected more DEGs in the branches than in the leaves. Branches connect leaves to roots. The phloem contains sugars, hormones, and other substances such as small RNAs (Lough and Lucas, 2006), which play a very important role in plant growth and signal transduction. Further experiments are required to explore how changes in gene expression affect the composition of substances in the branches and roots of *Rosa* plants under low-temperature stress.

### 4.3 Transcript profiles of cold tolerance-related genes in *R. hybrida*

Oxygen is involved in plant cell metabolism, but under low-temperature stress, the activity of enzymes related to metabolic activities decreases, and the oxygen utilization rate also decreases. This can lead to the accumulation of reactive oxygen species (ROS), which react with various cellular components because of their unpaired electrons and highly active state. This can result in abnormal cell metabolism and even death, thereby disrupting normal growth and development. The organs with more active metabolism produce more ROS. The antioxidant system can remove ROS and help to maintain the balance between their production and consumption (Dou, 2013). POD participates in the antioxidant system. Studies have shown that the activity and abundance of POD in grape branches increases after low-temperature acclimation, and these increases are greater in varieties with stronger cold resistance (Wang et al., 1996). In a study on winter wheat and spring wheat, Baek and Skinner (2003) found that at the early stage under a low-temperature treatment, there were increases in the transcript levels of genes encoding antioxidant enzymes such as mitochondrial manganese superoxide dismutase, cytosolic monodehydroascorbate reductase, chloroplast dehydroascorbate reductase, thylakoid-associated ascorbate peroxidase, and glutathione peroxidase. In this study, we found that *POD17* in *R. hybrida* was first down-regulated, then up-regulated, and then down-regulated under a 3-h freezing treatment. This may have increased the activity and/or abundance of POD around 2 h of freezing treatment, but further research is required to determine whether changes in gene transcript levels are consistent with changes in the levels of the corresponding protein.

*NDUFA9* encodes a subunit of NADH dehydrogenase, which is the first enzyme in the oxidative phosphorylation pathway. This pathway oxidizes organic matter such as sugars, lipids, and amino acids to release energy and generate ATP to fuel all of the essential processes for plant life. Low-temperature stress in plants affects the expression of mitochondrial genes encoding NADH dehydrogenase subunits, resulting in defects in germination, growth, and development, and other changes in response to hormonal changes (Zeng et al., 2018). In this study, we found that *NDUFA9* in *R. hybrida* was down-regulated and then up-regulated during a 3-h freezing treatment. These results imply that this gene plays a role in the late stage of the cold-resistance response of *R. hybrida*.

*PMA1* encodes an H^+^-ATPase. In plants, H^+^-ATPases participate in various processes, such as pH regulation within cells, cell elongation and growth, stomata opening and closing, and transmembrane transport. It is known as the “master enzyme” of essential plant processes (Yang et al., 2006). When cold-sensitive plants are subjected to low temperatures, the ATPase on the membrane is inactivated and its activity decreases, which weakens active transport, increases membrane permeability, and results in the passive export of potassium ions and sugars. The plasma membrane H^+^-ATPase activity of eggplant leaves was found to double under mild freezing stress, but it decreased under more severe freezing stress. In the needles of very cold-hardly pine trees under severe freezing stress, the plasma membrane H^+^-ATPase activity was found to be significantly increased (Mao et al., 2003). In this study, *PMA1* was gradually up-regulated in *R. hybrida* during the 3-h freezing treatment. This implies that the abundance of the proton transport ATPase increased to maintain active cell transportation, indicative of the low-temperature adaptability of this material.

Sugars function as osmotic regulation substances in plants, and their metabolism is greatly affected by low temperature. The accumulation of soluble sugars can alleviate the osmotic stress caused by low temperature and improve cold resistance (Chen, 2012). β-amylase catalyzes the formation of maltose from starch, and the accumulation of maltose helps to protect the electron transport chain and proteins in the chloroplast matrix under low-temperature stress. In another study, knock-out of the *Arabidopsis* β-amylase *BMY8* gene blocked maltose accumulation, resulting the accumulation of starch. The freezing resistance of the knock-out line was significantly lower than that of the wild type, and its photosystem II was more sensitive to cold stress (Kaplan and Guy, 2005). In the present study, we found that *β-Amy1* in *R. hybrida* was first up-regulated, then down-regulated, and then up-regulated during a 3-h freezing treatment. We speculate that *β-Amy1* in *R. hybrida* was induced by low temperature at the initial stage of stress. This would increase starch degradation, leading to the accumulation of maltose, and consequently, less cell damage. As the duration of the freezing treatment extended, the tolerance limit was gradually reached and *β-Amy1* was down-regulated, so that the maltose content decreased. Thus, we speculate that this gene plays a very important role in the early stages of the response to freezing.

The expression of genes related to cold tolerance in plants is affected by multiple factors such as the duration (time) and intensity (temperature) of the cold treatment. Further studies should test the effects of cold treatments of different duration and intensity on gene expression in *Rosa.*

## 5 Conclusion

Nine *Rosa* materials were subjected to a low-temperature treatment to identify their degree of cold tolerance. *R. hybrida, R. rugosa ‘Pingyin 12’*, and *R. rugosa* were selected as cold-resistant materials, that enriched the resources of high-quality cold resistant varieties, provided scientific basis for cold resistance breeding and cultivation management, and expanded the cultivation and distribution range of *Rosa* plants. We screened the transcriptome data to identify DEGs related to cold tolerance in *R. hybrida*. GO enrichment analysis revealed that genes showing significant changes in their transcript levels under short-term low-temperature stress were involved in sugar metabolism. The KEGG pathway enrichment results showed that 47 significant DEGs under low temperature were enriched in 57 pathways, Among them, Carbohydrate metabolism and Signal transduction were classified with the largest number of enrichment pathways The transcript profiles of four cold tolerance-related genes in *R. hybrida* were determined by qRT-PCR. The four genes, *POD17*, *NDUFA9*, *PMA1*, and *β-Amy1*, showed different expression patterns during a 3-h freezing treatment. All four of these genes were involved in the low temperature response in *R. hybrida.*

Plant cold resistance is the result of genetic factors and environmental factors, so it needs to be studied from various angles and by various methods. Although the selection of suitable planting land, increasing soil fertility and improving light and temperature conditions can improve the cold resistance of plants, the research on the molecular mechanism of cold resistance can lay the foundation for molecular breeding for cold resistance. Low temperature is a kind of abiotic environmental stimulus. In order to adapt to environmental changes, plants will produce various physiological responses and their own defense systems to resist cold and freezing. In this study, cold tolerance identification of *Rosa* materials and expression patterns of genes will lay a foundation for cold-resistant molecular breeding and mechanism analysis.

## Data availability statement

The original contributions presented in the study are included in the article/**Supplementary Material**. Further inquiries can be directed to the corresponding authors.

## Author contributions

XC, HW, QS, JX: Conceptualization, Methodology, Formal analysis, Resources, Writing-Reviewing and Editing, Funding acquisition. QS, HW: Investigation, Data Curation, Writing-Original draft preparation. DC: Writing-Reviewing, Validation. CL: Writing-Reviewing. HL: Methodology, Supervision. CH: Supervision, Funding acquisition, Project administration. All authors contributed to the article and approved the submitted version.
