# Supplementary material for: Cold tolerance identification of nine Rosa L. materials and expression patterns of genes related to cold tolerance in Rosa hybrida
Source: Front Plant Sci. 2023 Jun 27;14:1209134. doi: 10.3389/fpls.2023.1209134 (PMC10333502; doi:10.3389/fpls.2023.1209134)
Supplement: Supplementary file 1 [file DataSheet_1.zip › Figure S3.PDF]

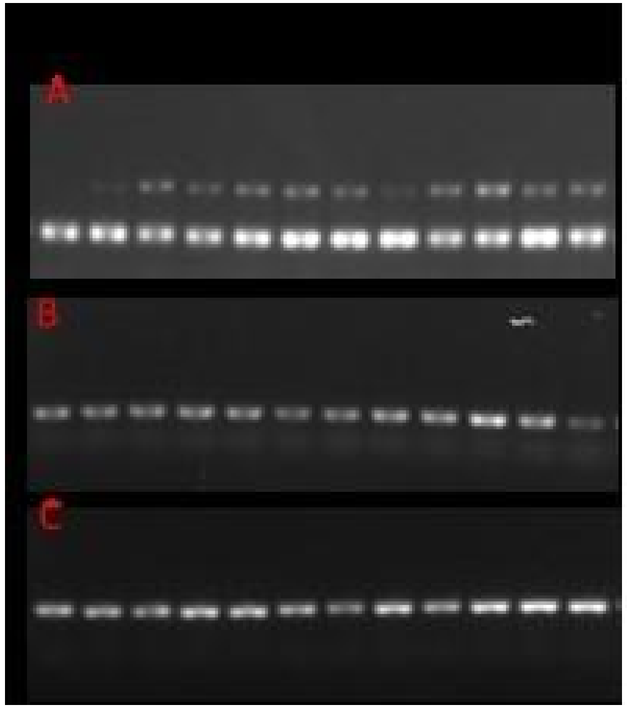

Semi quantitative of 3 reference genes

Note: Fig A, B, C are gelograms of *GAPDH*, *ACT4*, *UBI1*.

Supplemental Figure 3 Semi quantitative of 3 reference genes
