# Supplementary figures and images for: Cold tolerance identification of nine Rosa L. materials and expression patterns of genes related to cold tolerance in Rosa hybrida
Source: Front Plant Sci. 2023 Jun 27;14:1209134. doi: 10.3389/fpls.2023.1209134 (PMC10333502; doi:10.3389/fpls.2023.1209134)

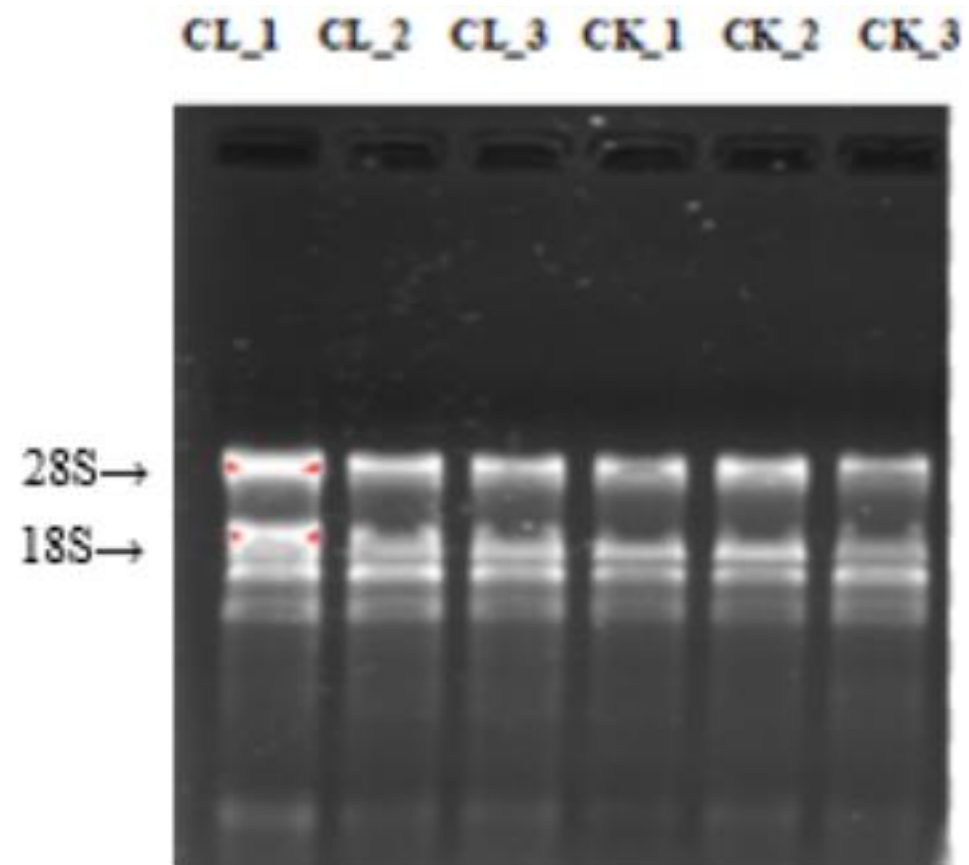

Supplemental Figure 1 Gel electrophoresis of sample RNA in RNA-Seq

Supplement: Supplementary file 1 [file DataSheet_1.zip › Figure S1 (2).PDF]

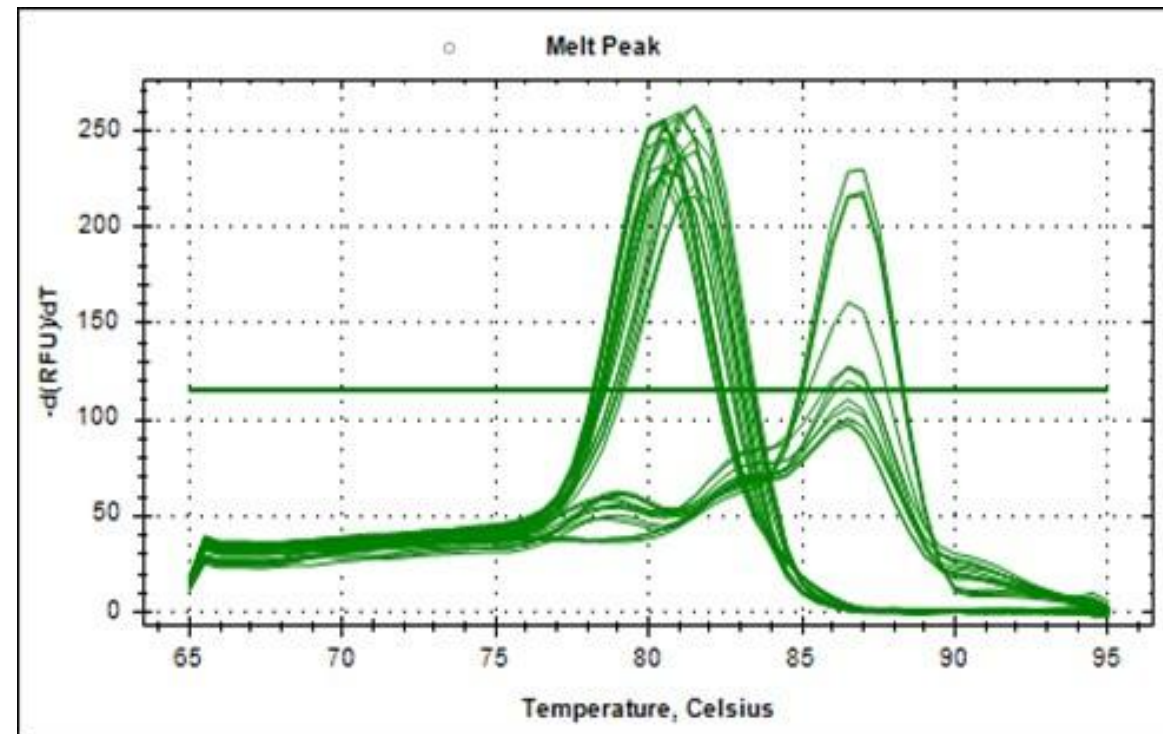

Supplemental Figure 2 Quantitative detection of cDNA library concentration by qPCR

Supplement: Supplementary file 1 [file DataSheet_1.zip › Figure S2 (2).PDF]
